# Disease Extent ≥4 cm Is a Prognostic Marker of Local Recurrence in T1-2 Breast Cancer

**DOI:** 10.4061/2011/860584

**Published:** 2011-08-10

**Authors:** D. Lindquist, D. Hellberg, T. Tot

**Affiliations:** ^1^Department of Pathology and Clinical Cytology, Central Hospital Falun, SE-791 82 Falun, Sweden; ^2^Center for Clinical Research Dalarna, Uppsala University, Nissers väg 3, 791 82 Falun, Sweden

## Abstract

Despite improvements of the therapy for breast cancer, a proportion of the patients still get local recurrence. The status of the surgical margins is the most often used parameter for decision regarding additional treatment. However, a negative margin is not a guarantee that there is not residual cancer left in the breast; additional parameters are needed to better predict the risk of local recurrence. The disease extent was evaluated in the surgical specimen from 313 women after breast-conserving therapy using large-section histology and was correlated to the incidence of local recurrence. A disease extent ≥4 cm was shown to be an independent marker for local recurrence; the cumulative 10-year local relapse rate for the group with a disease extent ≥4 cm was 20.5%, and for the rest 6.7%. We conclude that disease extent ≥4 cm seems to be an important factor when evaluating the risk for local recurrence.

## 1. Introduction

Breast cancer is the most common type of malignancy in women and the most common cause of cancer-related death in women. Almost 1.4 million new cases are diagnosed in the world each year, and approximately 458.000 women die from the disease every year [1]. Several clinical trials have established the adequate surgical treatment being either breast-conserving therapy combined with radiotherapy, or radical mastectomy [2, 3]. Although these surgical procedures assure successful local control of the disease in the majority of the cases, a considerable proportion of patients still get local recurrence. The cumulative incidence of local recurrence in two trials after 20-year follow-up was 8.8% and 14.3% in women treated with breast-conserving therapy combined with radiotherapy [2, 3]. Some reports indicate that this incidence is declining, mostly due to increasing use of adjuvant hormonal therapy or chemotherapy [4]. 

The presence of positive surgical margin is an important factor when evaluating the risk for local recurrence, but there is no clear consensus on the definition of negative margins, and the need for a completing surgical intervention when the margins are positive is under debate [5]. This question is discussed by Morrow, including the statement that “residual breast cancer is present in the breast in 32–63% of women with clinically and mammographically unicentric tumors, regardless of the margins of excision are positive or negative” [6]; therefore the need for a new surgical intervention is unclear [5, 6]. Local recurrence may develop after excision of a tumor with negative surgical margins indicating that in these women breast-conserving therapy and irradiation may not be the adequate treatment and that local recurrence cannot be properly predicted based on the single parameter of surgical margin status. 

Other parameters which have been tested with regard of predicting local recurrence are patient age, lymphovascular invasion, tumor size, tumor grade, hormonal therapy, and chemotherapy [7]. The prognostic value of the histological extent of the disease and distribution of the lesions has rarely been assessed, mostly because it requires special histopathological techniques. Large section histology is used routinely in only few pathology laboratories when examining the breast specimen [8, 9], but this should not limit the use of the valuable results generated with such approach. Early studies on whole-organ histopathology already indicated that a substantial proportion of breast carcinomas are extensive and multifocal [10, 11] and that these factors are prognostic markers [10]. This is supported by studies on modern breast imaging [12]. Some histopathological studies have not shown such correlation [13, 14], although these studies did not use large-section histology. Recently, the disease extent was shown to correlate with presence of lymphovascular invasion and lymph node status [9]. Other studies have evaluated the distribution of lesions as markers for lymphovascular invasion and lymph node status, using large-section histology [15, 16]. 

The aim of this study was to evaluate the disease extent, determined with large-section histology, as a prognostic marker for local recurrence in patients treated with breast-conserving surgery, and in addition, to find an appropriate cutoff for defining extensive disease.

## 2. Materials and Methods

### 2.1. Patients and Material

A total of 313 patients were included in the study after approval from The Regional Ethical Review Board in Uppsala, and the study was conducted in accordance with the Declaration of Helsinki. The study material was collected prospectively at the Department of Pathology and Clinical Cytology, Falun Central Hospital, Sweden and included all patients with invasive or in situ carcinomas of the breast that were treated with breast-conserving surgery, and had a measurable disease extent upon histological analyses during 1996–1998. During this time period, 586 women were diagnosed with breast cancer in the county of Dalarna with a population of approximately 250.000. Of these 586 women, 229 (39%) were treated with mastectomy, 321 (55%) were treated with breast-conserving therapy, and 36 (6%) either refused or could not be offered surgical treatment. All patients who primarily were treated with breast-conserving therapy but were later offered an additional mastectomy due to margin status, tumor size, or multifocality, were not included in the present study. Of the 321 patients given breast conservative therapy only, 8 did not have a measurable disease extent, due to technical reasons, and were thus excluded from the study. Data regarding treatment and follow-up was reported by the surgeons at the tumor board meeting or collected from patient files. Patient and tumor characteristics are shown in Table 1. 

### 2.2. Histopathological Preparation and Evaluation of Disease Extent

All surgical specimens were worked up using large-section histopathology technique, which has been a routine procedure at our department since 1982. The method has been described in detail previously [17, 18]. In short, all cases were discussed by a preoperative tumor board, where the radiological extent and distribution were registered. Postoperatively, the whole sector resection specimens and 3–4 mm tissue slices from the sector resection cut parallel to the pectoralis fascia were radiographed. The most representative slices were selected and embedded into separate large-section blocks. The selection was based on previous radiological findings, and all lesions detected by the radiological examinations were included in the embedded section. Thus, no lesions detected by radiology were missed, but new lesions were frequently observed. Margin status was always assessed, and most patients with positive margin status were offered additional surgery, and the specimen from the second resection was added when the disease extent was determined as described below. 

**Figure 1:**
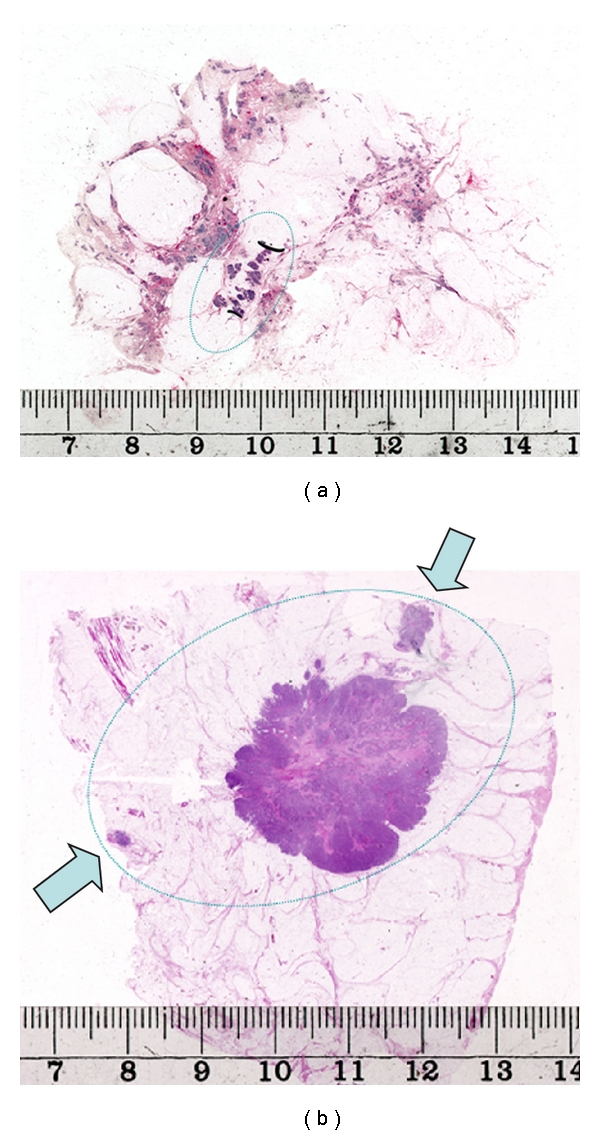
Examples of a breast carcinoma of limited extent (a) and a breast cancer with a disease extent ≥4 cm, with a tumor size smaller than 4 cm (b).

The disease extent was defined as the area of breast tissue involved in malignant structures, including invasive, in situ, and intravascular tumor structures that were observed in the large histological sections. These data were collected after routine histopathological evaluation and discussed on the postoperative tumor board meeting to be correlated with the radiological findings. This histopathological evaluation also included routine parameters, such as histological grade and tumor size. The criteria for disease extent, when having 4 cm as the cutoff, were identical to those in a previous study where disease extent was evaluated as a marker for lymphovascular invasion and lymph node metastasis [9]. Typical examples of breast carcinoma of limited extent and extensive breast carcinoma are shown in Figure 1.

### 2.3. Statistical Analysis

The different sizes of disease extent were evaluated as prognostic markers for the risk of local recurrence using the Kaplan-Meier method and the log-rank test to compare the distributions. When significant differences were found, a Cox Regression multivariate analysis was performed, including known risk factors for local recurrence and differences in treatment, to compare the distributions with possible confounding factors. Local recurrence was defined as the event, and patients dead for another reason or patients who moved out of Dalarna and thus were lost to follow-up, were censored at last follow-up, free of local recurrence. To assess whether age and follow-up time equally distributed among patients with extensive and nonextensive tumors, a Mann Whitney test was used. A chi-square test was used to test the sampling distribution for ordinal variables. All significance testing was performed at the 0.01 level.

## 3. Results

**Figure 2:**
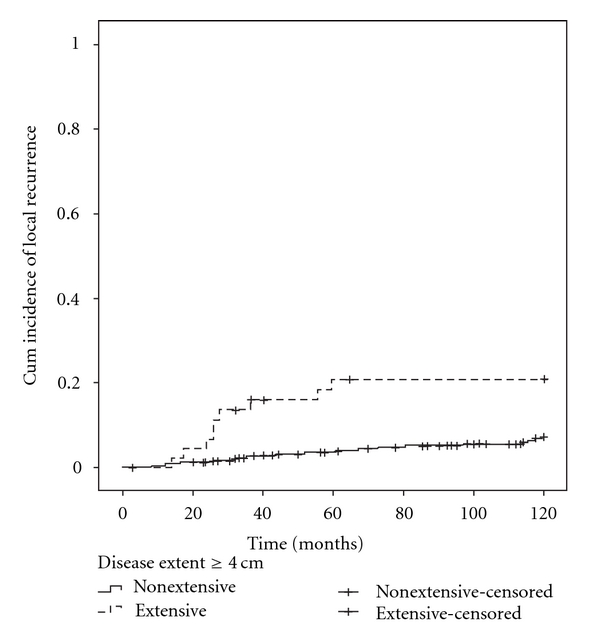
The risk of ipsilateral recurrence is significantly higher for patients with extensive tumors more than 4 cm (*P* = 0.001, log rank test).

Three different cutoff values for disease extent, 4 cm, 3 cm, and 2 cm, were tested. Tumors with an extent larger than the cutoff levels were considered extensive, and the rest were considered nonextensive (or of limited extent). The differences in incidence of local recurrence were significantly higher for the patients where tumors ≥4 cm were considered as extensive tumors (*P* = 0.001, log-rank test), whereas the other levels of disease extent were not (*P* = 0.041 and *P* = 0.094, resp.). The graph illustrating the cumulative incidence of local recurrence, where tumors ≥4 cm were considered as extensive, is shown in Figure 2. Extensive and nonextensive tumors with 4 cm as the limit were then further analyzed in a Cox proportional multivariate analyzes, controlling for grade of the invasive component, grade of the in situ component, size of the primary tumor (>15 mm or ≤15 mm), age, if the patient were treated with irradiation, whether or not the patient were given hormonal therapy, if the patient had chemotherapy or not, and whether the patient where positive or negative for estrogen receptor staining and progesterone receptor staining. The difference remained statistically significant, and the detailed data is presented in Table 2.

Since postoperative irradiation is standard treatment after breast-conserving surgery today, the same analysis was also performed including only patients that were treated with irradiation, and the difference in risk of local recurrence remained statistically significant (*P* = 0.003, HR 12.06, CI 1.41–103.01). The 206 patients treated with radiotherapy were given a total dose of 56 Gy. Differences in distribution of patient and tumor characteristics were tested using either a chi-square test or a Mann Whitney test as illustrated in Table 1. Tumor grade for the in situ component and presence or absence of local recurrences were significantly different (*P* = 0.005 and *P* = 0.003, resp., chi-square test). 

Difference in tumor size was also assessed, and the distribution is shown in Table 1, where no statistically different distribution for T1 and T2 tumors could be observed. No invasive tumors had a higher T-stage than T2, according to the TNM classification system, and the largest tumor was 35 mm. Chemotherapy was not given regularly during the time period studied; 10 patients were treated with FEC and 12 patients with CMF; due to the small number of patients they were combined as one group in the analyses. For those patients treated with hormonal treatment, 51 patients had tamoxifen, and 1 patient had aromatase inhibitor, due to adverse effects from tamoxifen. If the patient who was recommended to take tamoxifen by the physician and stop doing so after a while, a minimum of 3 years of treatment were used as a cutoff when collecting data; thus all 51 patients had taken tamoxifen for at least 3 years.

## 4. Discussion

In this study a disease extent ≥4 cm, evaluated on large-section histology, was shown to be a prognostic marker for local recurrence in women treated with breast-conserving surgery, a finding not previously reported. Large-section histology has shown to be superior to conventional histology technique in determining morphologic parameters of prognostic value, and a better disease-specific survival has been reported for patients with unifocal tumors compared with both tumors of multifocal and diffuse distribution [19]. There are other studies reporting a better survival for patients with unifocal tumors [20], whereas others found no such correlation [21]. Distribution of lesions evaluated by large-section histology has also shown to be correlated with lymphovascular invasion and lymph node metastasis [15, 16]. 

When reporting distribution of lesions and disease extent there is a discrepancy between definitions, sometimes only the invasive foci are considered, resulting in a lower proportion of multifocal disease and a smaller disease extent, as compared with our definitions. When measuring the histopathological disease extent with conventional histopathology, small or microscopic lesions might be missed at macroscopy and radiology, and when the specimen is fragmentized, the relationship between multiple tumor foci will be more difficult to evaluate in a standardized way. There is obviously a need for standardization of how to define multifocal tumors and disease extent, to be able to compare these findings. Large-section histology is an excellent tool for standardizing evaluation of breast cancer specimen, combined with radiological-pathological correlation, and a cost-benefit analysis for using this technique has recently been published [22], demonstrating that the cost is not higher as compared to how the analysis is performed in many other places, except for if the analysis is inadequate. Importantly, all histopathological parameters routinely assessed, including evaluating margin status, can also be determined with large-section histology. 

The findings in this study clearly demonstrate that a disease extent ≥4 cm is an independent marker for local recurrence in breast cancer patients. When patients are treated with breast-conserving therapy, the cancer can still be multifocal, and since there is by definition normal tissue between the foci, smaller foci might be left in the breast, despite negative margins when examining the surgical specimen, as discussed in detail elsewhere [5, 6]. The question is then whether treatment with radiotherapy, or other adjuvant treatment, is enough to cure the women [6]. Obviously, there is a group of women that develop local recurrence and may have benefited from more aggressive surgery as primary treatment. Disease extent, evaluated by large-section histology, is thus an important marker for risk of local recurrence; it should be evaluated further, as it might be useful in clinical practice when treatment strategy is discussed. All patients in this study had tumors no larger than T2 (the largest T2 tumor was 35 mm in diameter). A tumor size larger than 4 cm is often the maximum size for breast-conserving therapy recommended by most guidelines. However, in this study, 44 tumors had a disease extent which was larger than 4 cm but obviously a tumor size smaller than 4 cm, and in this group 9 patients (20%) had a local recurrence. Perhaps it is not tumor size, but the total area over which the tumor has been spread, regardless whether there is normal tissue between the foci, which should be taken into consideration when determining if the patient should be offered an additional mastectomy or not. 

In this study the cumulative incidence of local recurrence for patients with extensive tumors was more than 20% which is high, but this also includes a proportion of patients who were not given postoperative radiotherapy (which was usually offered within clinical trials at the time these women were diagnosed). When studying only the group of patients that were given radiotherapy, the cumulative incidence of local recurrence was 11% for patients with extensive disease and 3.4% for patients with nonextensive disease, which is in line with previous observations [2–4], and the difference in distribution between the groups was still significantly different. There was however no difference in the distribution of the groups that were given radiotherapy or not, when comparing extensive and nonextensive tumors (Table 1). The only parameter which was different when comparing the extensive and nonextensive tumors was the grade of the in situ component, which is known to be a risk factor for local recurrence [7]. This factor was included in the multivariate analyses, and the difference in incidence of local recurrence between extensive and nonextensive tumors remained significantly different. 

The major limitation of this study is of course that it is retrospective and also that the guidelines for neoadjuvant treatment are different compared to what is usually offered today. However, no differences were observed between the patients with extensive and nonextensive tumors, when comparing radiotherapy, hormonal therapy, and chemotherapy (Table 1). The large-section histology method is one of the strengths in this study; another important factor is the long follow-up time (which is the obvious reason why the treatment guidelines were differently compared with today) and the possibility to include all patients that had been treated with breast-conserving therapy in the county of Dalarna during a 3-year time period, with a 10-year follow-up. Only 8 patients (2.5%) were excluded due to technical reasons, a figure far lower than several studies. The minimum follow-up of 3 months' time illustrates patients dead for another reason or patients who moved elsewhere and thus were lost to follow-up. However, the first quartile of follow-up time is 120 months, indicating that at least 75% of the patients had been followed for this time. 

The cutoff value of 4 cm for disease extent has been used in one previous study [9] and was then chosen based on the average size on the specimen. Approximately 6–7 cm is the average width of surgical specimen in our material which allows radical excision of a tumor of 4 cm with appropriate circumferential margins, and tumors of limited extent (less than 4 cm) are thus good candidates for breast-conserving surgery. For the extensive tumors, where the risk of local relapse is considerably higher, a modified radical mastectomy might be considered, to spare the patient from multiple operations and possibly irradiation. This is however not applicable in all cases, since the size of the breast also is important when determining the type of surgical treatment; thus larger tumors can sometimes be offered breast-conserving treatment if the breast is large. No such data was possible to collect in this study, but this should not be a very frequent approach.

Data from this retrospective study could probably not be used to change guidelines for treatment but illustrates that assessing the disease extent with large-section histology is important to validate further, since the method also has shown to be useful to predict other important prognostic factors. This includes a correlation between distribution of lesions or disease extent and presence of lymphovascular invasion and lymph node status [9, 15, 16], and a better disease-specific survival has been reported for patients with unifocal tumors, compared with both tumors of multifocal and diffuse distribution [19]. The importance of large-section histology is further emphasized by the fact that a cost-benefit analysis has demonstrated that the method does not increase the cost [22] but also allows for the possibility to evaluate all other parameters usually assessed, including margin status. Thus, in the era where several new and expensive biological markers are emerging, in this study we demonstrate a histological method, where several prognostic markers show promising value, without any additional cost. Therefore, the authors suggest that the method of large-section histology should be used and evaluated more frequently.

## 5. Conclusion

This study demonstrates the usefulness of the large-section histology method to evaluate disease extent and demonstrate that a disease extent ≥4 cm is a prognostic marker for local recurrence in women with breast cancer.

## Figures and Tables

**Table 1:** Patents and tumor characteristics.

Characteristic	All	Extensive	Non extensive	
		≥4 cm	<4 cm	*P* value^a^
Number of patients	313	44	269	
Age				
Median	61.2	59.4	61.5	0.162
Mean	61.0	58.7	61.4	
Disease extent				
≥4 cm	44	44		
≥3 cm	36		36	
≥2 cm	70		70	
<2 cm	163		163	
Size of dominating tumor mass				
≥15 mm	180	29	151	0.224
<15 mm	133	15	118	
T-classification				
T1	211	22	189	0.045
T2	36	8	28	
T3 or T4	0	0	0	
Local recurrence				
Yes	27	9	18	0.003
No	276	35	251	
Follow-up time (months)				
Median	120	120	120	0.108
Mean	106	95	108	
Min	3	14	3	
Max	120	120	120	
Grade of invasive lesion				
I	93	10	83	0.900
II	107	13	94	
III	45	6	39	
Missing	68	15	53	
Grade of in situ lesion				
I	109	16	93	0.005
II	99	8	91	
III	59	16	43	
Missing	46	4	42	
Radiotherapy				
Yes	206	28	178	0.736
No	93	14	79	
Missing	14	2	12	
Hormonal therapy				
Yes	52	11	41	0.101
No	196	24	172	
Missing	65	9	56	
Chemotherapy				
Yes	22	3	19	0.946
No	226	32	194	
Missing	65	9	56	
Estrogen receptor status				
Positive	215	26	189	0.704
Negative	28	4	24	
Progesterone receptor status				
Positive	171	22	149	0.759
Negative	70	8	62	

^a^Test of variable distribution between patients with extensive versus nonextensive tumors, using either a Mann Whitney test or a chi-square test where appropriate.

**Table 2:** A disease extent ≥4 cm is the only significant prognostic marker for an increased risk of local recurrence.

	HR^1^	(99% CI^2^)	*P*-value
Disease extent ≥4 cm	11.099	(1.264–97.464)	0.004
Size ≥15 mm or <15 mm	2.699	(0.392–18.596)	0.185
Grade of in situ lesion	0.350	(0.051–2.403)	0.160
Grade of invasive lesion	1.584	(0.165–15.165)	0.600
Radiotherapy	0.273	(0.021–3.577)	0.194
Hormonal therapy	0.000	(0.000–<100000)	0.959
Chemotherapy	2.618	(0.082–83.180	0.474
Estrogen receptor status	462417	(0.000–<100000)	0.982
Progesterone receptor status	0.568	(0.057–5.657)	0.526
Age	0.958	(0.868–1.058)	0.267

^1^Hazard Ratio, ^2^Confidence Interval.
